# Comparative study between efficacy of dexamethasone-prostaglandin-receptal combination and mechanical correction in uterine torsion cases in Egyptian buffalo-cows (*Bubalus bubalis*)

**DOI:** 10.1186/s12917-023-03651-y

**Published:** 2023-07-24

**Authors:** Arafat Khalphallah, Enas Elmeligy, Taher Al-Daek, Hassan A. Hussein, Ragab H. Mohamed, Mahmoud S. Sabra, Asem Mohammed Zakaria, Marwa I. Khalifa, Haitham H. Mohammed, Khaled. A. Khesruf, Rezk Said Ghallab

**Affiliations:** 1 Division of Internal Medicine, Department of Animal Medicine, Faculty of Veterinary Medicine, Assiut University, Assiut, 71526 Egypt; 2 Veterinary Teaching Hospital, Faculty of Veterinary Medicine, Assiut University, Assiut, 71526 Egypt; 3 Faculty of Veterinary Medicine, Omar Almukhtar University, Bayda, Libya; 4 Department of Theriogenology, Faculty of Veterinary Medicine, Assiut University, Assiut, 71526 Egypt; 5 Department of Theriogenology, Obstetrics, and Artificial Insemination, Faculty of Veterinary Medicine, Aswan University, Aswan, 81528 Egypt; 6 Department of Pharmacology, Faculty of Veterinary Medicine, Assiut University, Assiut, 71526 Egypt; 7 Department of Food Hygiene, Faculty of Veterinary Medicine, Aswan University, Aswan, 81528 Egypt; 8 Department of Rangeland, Wildlife and Fisheries Management, Texas A&M University, College Station, TX 77843 USA; 9 Department of Animal diseases, Faculty of Veterinary Medicine, Aleppo University, Aleppo, Syria; 10 Department of Theriogenology, Faculty of Veterinary Medicine, Matrouh University, Matrouh, 51744 Egypt

**Keywords:** Buffalo-cows, Clinical findings, Dexamethasone-prostaglandin-receptal combination, Placental characterization, Somatic cell count, Uterine torsion

## Abstract

**Background:**

According to reports, the majority of domesticated species exhibited uterine torsion. It was occasionally noted as a cause of dystocia in buffaloes. The uterus might twist more frequently late in pregnancy because of certain animal traits. The current research monitored the clinical findings and laboratory assays associated with uterine torsion cases in pregnant buffalo-cows through comparing between normal labored buffalo-cows (Norm-Lab^gr^; n = 20), mechanically corrected uterine torsed animals without medicament interference (UtrTors^gr^; n = 160), and mechanically corrected uterine torsed animals with medicament interference (UtrTors-Med^gr^; n = 40) through focusing on placental characterization, calves body weight, milk constituents and milk somatic cell count (SCC) in normal labored buffaloes and uterine torsed ones. Through clinical and laboratory investigations of these buffaloes (N = 220) had been conducted 3 times; 7 h pre-calving and post calving (Post uterine correction) i.e. 48 and 96 h. Uterine torsion prevalence parameters, placental characterization, calves body weight, milk constituents and milk somatic cell counts were evaluated in normal labored buffaloes and uterine torsed ones.

**Results and Conclusions:**

The study concluded pre-calving remarkable variations in clinical findings, leukogram picture, calf birth weight and some placental characterization parameters between Norm-Lab^gr^ and each of UtrTors^gr^ and UtrTors-Med^gr^ whereas these variations disappeared post-partum as a result to either only mechanical correction or mechanical correction plus medicaments interference. No pre-or post-calving significant changes between UtrTors^gr^ and UtrTors-Med^gr^ except for the abnormal clinical findings were more representative in UtrTors-Med^gr^ than those in UtrTors^gr^ particularly pre-calving. The applied pre-calving therapeutic regimen including dexamethasone-prostaglandin-receptal combination had a powerful potential efficacy that induced vaginal delivery of calves in UtrTors-Med^gr^ as well as prepartum mechanical correction of torsed uterus approved higher efficacy in UtrTors^gr^. The applied prepartum mechanical correction of torsed uterus and/or pre-calving therapeutic regimen as well as subsequent post-calving, post uterine correction applied medicament treatment accelerated rapid recovery of affected buffalo-cows through achieving rapid restoring of their physiological parameters. Buffalo-cow’s milk composition, milk pH and milk SCC were not affected whereas no significant variations were reported between Norm-Lab^gr^, UtrTors^gr^ and UtrTors-Med^gr^.

## Background

According to Purohit et al. [1], uterine torsion was the term used to describe the pregnant uterus twisting along its long axis, with the anterior vagina immediately caudal to the cervix acting as the point of torsion (post cervical torsion). Pre-cervical torsion, which occurred less frequently, was the site of torsion cranial to the cervix [2]. Ahmed et al. [3] mentioned that Boutrolle first described the disease in 1766.

Nanda and Sharma [4]; Ali et al. [5] reported strong association between uterine torsion incidence in large animals and advanced pregnancy as well as parturition. It had different incidences depending on the animals’ geographic distribution. According to reports, uterine torsion accounts for 67–83% of all dystocia cases in buffaloes [6].

The occurrence of uterine torsion and the timing of its occurrence in cattle underlined its effect on the health of the dam and, consequently, the economics of the dairy herd [7]. Additional diseases attributed to it included haemoperitoneum [8], ovarian vein rupture [9], rotation of the urinary bladder [10], intestinal blockage [11], and uterine perforation [12].

Most domesticated species were said to exhibit uterine torsion. As a cause of dystocia, it was infrequently noted in buffaloes [5;6], cattle [13], bitches [14], camels [15, 16], queens [17], ewes [18], goats [19] and mares [20], but rarely in sows [21]. The last trimester of pregnancy was when bovines were most vulnerable to uterine torsion [22]. Uterine torsion was revealed to be the cause of around 53–83% of the dystocia cases in buffaloes [23].

Although the precise reason of uterine torsion is not entirely understood, it had been noted that different phases of pregnancy got an impact on its occurrence. A high incidence was noted in the final stages of pregnancy, just before delivery [24]. Although severe tension could excite the vaginal stretch receptor, causing reflex abdominal straining, the high degree of torsion increased the likelihood of straining [22]. Because the majority of the time either the foetal limbs or the foetal membranes failed to reach the anterior vagina, there might be no straining [25].

Serious economic losses resulted from uterine torsion. Losses due to decreased milk production, foetal mortality and management of subsequent disorders, endometritis, prolonged uterine involution, and infertility were among them. Moreover, uterine torsion might be associated with the rotation of the bladder, ovarian vein rupture, haemoperitoneum, intestinal blockage, uterine perforation, and the development of adhesions between the uterus and the viscera around it [26].

A lot of inherited and non-inherited factors affected milk yield and composition in dairy animals, as lactation number, feeding, pregnancy status, dystocia, age, temperature, humidity, etc., uterine torsion considered as one of non-inherited factors that had great impact on drop in milk yield [27].

According to the authors knowledge, Pathological changes in the uterus, following uterine torsion were studied [28]. Also its effect on milk yield was reported by Berry et al. [29]; Gaafar et al. [30]. But pathological effect of uterine torsion on the placenta and its effect on the milk composition and somatic cell count in buffalo was not studied sufficiently, so the aim of the present study to monitor the clinical findings and laboratory assays associated with uterine torsion cases in pregnant buffalo-cows (*Bubalus bubalis*) through comparing between normal labored buffalo-cows (Norm-Lab^gr^), mechanically corrected uterine torsed animals without medicament interference (UtrTors^gr^) and mechanically corrected uterine torsed animals with medicament interference (UtrTors-Med^gr^) through focusing on placental characterization, calves body weight, milk constituents and milk somatic cell counts in normal labored buffaloes and uterine torsed ones.

## Materials

### Animals

A total number of 220 late pregnant buffalo-cows (*Bubalus bubalis*) of age ranged between 5 and 8 years (380–450 kg) were involved in this study. The farm’s system was free stall. These animals were selected from private farms in Assiut, Sohag and Aswan Governorates, Egypt in the period from 2015 to 2020. Their body weights ranged between 380 and 450 kg. Total mixed ration (TMR) was their main feed. Out of the 220 pregnant buffaloes, 20 animals had normal vaginal delivery while the other 200 buffalo-cows had uterine torsion that either responded to mechanical correction of torsed uterus and thus was named UtrTors^gr^, or did not respond to this mechanical interference and required medicament interference to enhance vaginal delivery of buffalo-calves and thus was named UtrTors-Med^gr^. Accordingly, the pregnant buffaloes (n = 220) were classified into 3 groups; normal labored buffalo-cows (Norm-Lab^gr^; n = 20), mechanically corrected uterine torsed animals without medicament interference (UtrTors^gr^; n = 160) and mechanically corrected uterine torsed animals with medicament interference (UtrTors-Med^gr^; n = 40). These animals were clinically and laboratory examined and sampled mainly three times; 7 h pre-calving (Before uterine correction and/or medicament interference) and post-calving i.e. 48 and 96 h (Following uterine correction and/or medicament interference).

### Samples

A whole blood sample was collected on ethylenediamine tetra acetic acid and stored at 4 °C until analysis. Blood serum samples were collected on plain vacutainer tubes and stored at − 20 °C until analysis according to Coles [31].

### Clinical examination

All late pregnant buffalo-cows underwent a thorough clinical examination as described by Cockcroft [32]. Clinical examination including estimation general signs of health such as temperature, pulse rate, respiratory rate, rumen movements, mucous membranes, lymph nodes, and skin. Digestive system and cardiothoracic organs were also clinically assessed. All Animals were introduced during late stage of pregnancy (9th month) till full term with history of colicky pains, depression, anorexia and some animals came in recumbent position with more or less tympani. After 72 h on onset of calving, disappearance of the signs of labor pain is normal irrespective of expulsion of calf. All animals had calving at full term either normal or needed interference.

Presence of torsion were confirmed through the vaginal and rectal examination according to [5, 25]. The location of broad ligaments or the twist in the vagina was the basis to determine the degree (sever, moderate or mild), The direction of the uterine torsion (clock-wise or anticlock-wise) as described by [5, 26].

### Complete blood count

Various hematological indices were manually measured as they included complete blood picture i.e. red blood corpuscles (RBCs), total leucocytic count (TLC), differential leukocytic count (DLC), haemoglobin (Hb) and packed cell volume (PCV). DLC was determined according to Coles [31]; Harvey [33]; Latimer et al. [34].

### Treatment strategies

The treatment of buffalo-cows with uterine torsion was firstly carried out through mechanical method (Rolling technique with blank) according to Zaher et al. [25]. The diseased cattle were manually and medicinally treated after clinical examination, and whole blood samples collection was conducted for each cow according to Claydon [35]; Lewing et al. [36]; Shukla et al. [37]. The uterine torsed buffaloes that were not responded to mechanical rolling as a tool for correction of uterine torsion and in which the cervix was still closed, were subjected after 12 h following the mechanical treatment to the following therapeutic regimen to induce opening of the cervix by using single IM doses of the followings; 2 ml of cloprostenol (Estrumate® Prostaglandin 20ml i.e. 10-Dose Vial, MERCK Animal Health, Intervet Inc., USA) as a synthetic prostaglandin analogue structurally related to prostaglandin F2α (PGF2α), dexamethasone 5 (Dexamethasone sodium phosphate 5 mg/ml, Vetoquinol, Québec, Canada) at a dose of 20 mg/cow and 5 ml/cow of Receptal® (0.004 mg/ml Solution for injection, vial 10 ml i.e. 1 ml contains 0.0042 mg buserelin acetate equivalent to 0.004 mg buserelin, MSD Animal Health, Buckinghamshire, UK). After calving, all uterine torsed buffaloes were subjected to the following therapy; oxytetracycline HC tablets at dose1 tablets/50 kg B.W./12 hrs for 3 consecutive days as antibacterial drugs had been administered via the uterus (Terramycin®, each tablet contains 250 mg of oxytetracycline HCl, Pfizer Animal Health, Division of Pfizer Inc, New York, NY 10,017, USA). IV injection of marbofloxacin at dose 1ml/50kg/cow/q24h (Marbocyl™ 10% Solution for Injection, Vetoquinol UK Ltd, West Northamptonshire, England, UK) for successive 5 days. IV infusion of 500 mL of a 50% solution of glucose (5%® Fath for drug and cosmetics industry FIPCO, Cairo, Egypt) q12h for successive five days. IV injection of 30 ml clanobutin sodium/cow/q24h (Bykahepar®, Schering-Plough Animal Health; MSD Animal Health, Kenilworth, New Jersey; USA) for successive 3 days. Ringer-Lactate solution for I.V Infusion (Ringer-Lactate solution 500 ml for I.V Infusion BP 2015, Fath for drug and cosmetics industry FIPCO, Cairo, Egypt) at dose 500 mL/cow/q12h for successive 5 days. IV injection of 30 ml clanobutin sodium/cow/q24h (Bykahepar®, Schering-Plough Animal Health; MSD Animal Health, Kenilworth, New Jersey; USA) for successive 3 days. The feed additive, i.e., Smartamine (Smartamine® M, Kemin company, USA, imported by United Bio-med Co., Cairo, Egypt) in a dose of 15 g daily, per os, for successive 30 days. Smartamine included amino acids (methionine and lysine).

### Uterine torsion prevalence i.e. degree, rolling for treatment, duration and calves survival rates

Uterine torsion prevalence included degree of uterine torsion, numbers of rolling for treatment, torsion duration and calves’ survival rates were monitored for each animals according to Ali et al. [5]; Manning et al. [38]; Frazer et al. [39].

### Buffalo-calf birth weights and placental characterization parameters

Buffalo-calf birth weights (kg) and placental characterization parameters were evaluated post-calving in Norm-Lab^gr^, UtrTors^gr^ and UtrTors-Med^gr^ in buffalo-cows.

Placental characterization parameters included placental weight (Pl weight; kg), total cotyledon weight (TCW; kg), total cotyledon number (TCN), average large cotyledon length (ALCL; cm), average medium cotyledon length (AMCL; cm), average small cotyledon length (ASCL; cm), average large cotyledon width (ALCW; cm), average medium cotyledon width (AMCW; cm), average small cotyledon width (ASCW; cm), average large cotyledon depth (ALCD; cm), average medium cotyledon depth (AMCD; cm), average small cotyledon depth (ASCD; cm), : placental efficiency (PE) and cotyledon density (CD). PE = calf birth weight/placental weight per kg. CD = number of cotyledon/ placental weight per gram.

### Milk sampling

Milk samples, 500 ml each, were collected from Norm-Lab^gr^, UtrTors^gr^ and UtrTors-Med^gr^ in buffalo-cows. Three milk samples were collected from each animal at the days 15, 20 and 25 after labor. All milk samples were transferred within 1 h to laboratory in ice box and were frozen till be examined. Milk compositions were determined by Lactoscan according to Khalifa and Zakaria [40]. Milk samples were kept frozen tell be examined. Chemical milk constituents included protein, fat, lactose and total solids as well as milk pH were measured by Lactoscan SL according to Chappalwar et al. (40]. Somatic cell count (SCC) was measured automatically by somatic cell counter according to Kamal et al. [41].

### Statistical analysis

Data were analyzed using SPSS statistical software program for windows version 10.0.1 (SPSS Inc., Chicago, IL., USA). The obtained data were described as mean ± standard deviation (SD). The data obtained from the clinical findings, laboratory analyses, calves birth weight and placental characterization parameters were analyzed by general linear model repeated measures ANOVA and significance level of results was set at p < 0.05. The significance of differences was evaluated between the means at different sampling times (7 h pre-calving, 48 h post-calving and 96 h post-calving) within the same buffalo-cow groups (Norm-Lab^gr^, UtrTors^gr^ or UtrTors-Med^gr^) or between different buffalo- groups (Norm-Lab^gr^, UtrTors^gr^ and UtrTors-Med^gr^) at different sampling times (7 h pre-calving, 48 h post-calving or 96 h post-calving).

## Results

### Clinical findings

The buffalo-cows with normal labor had normal clinical findings such as normal appetite, mucous membranes and Capillary refill time (CRT). Other abnormal findings including fever (≥ 39.6 Ċ), polypnoea (≥ 30/m), tachycardia(≥ 90b/m), pain test (+ ve), emaciation, diarrhoea and/or melena, cough, suppurative nasal discharges, enlarged lymph nodes, abnormal heart and lung sounds, reduced rumen movements, injected eye capillaries, dehydration, and alopecia, were not reported in Norm-Lab^gr^ either 7 h pre-calving, 48 h post-calving or 96 h post-calving (Table 1).

**Table 1:** The common findings in Norm-Lab^gr^ (n = 20), UtrTors^gr^ (n = 160) and UtrTors-Med^gr^ (n = 40) in buffalo-cows

	Norm-Lab^gr^			UtrTors^gr^			UtrTors-Med^gr^		
	Pre-calving^*^	Post-calving^*^	Post-calving^**^	Pre-calving^*^	Post-calving^*^	Post-calving^**^	Pre-calving^*^	Post-calving^*^	Post-calving^**^
Fever (≥ 39.6 Ċ)	0 (0)^*^	0 (0)	0 (0)	120 (75)	40 (25)	0 (0)	40 (100)	10 (25)	0 (0)
Polypnoea (≥ 30/m)	0 (0)	0 (0)	0 (0)	120 (75)	40 (25)	0 (0)	40 (100)	10 (25)	0 (0)
Tachycardia(≥ 90b/m)	0 (0)	0 (0)	0 (0)	120 (75)	40 (25)	0 (0)	40 (100)	10 (25)	0 (0)
Normal appetite	20 (100)	20 (100)	20 (100)	40 (33)	120 (75)	160 (100)	0 (0)	10 (25)	40 (100)
CRT (1–2 s)^43^	20 (100)	20 (100)	20 (100)	40 (33)	120 (75)	160 (100)	0 (0)	10 (25)	40 (100)
Pain test (+ ve)	0 (0)	0 (0)	0 (0)	0 (0)	0 (0)	0 (0)	0 (0)	0 (0)	0 (0)
Colicy pain signs	0 (0)	0 (0)	0 (0)	120 (75)	40 (25)	0 (0)	40 (100)	10 (25)	0 (0)
Emaciation	0 (0)	0 (0)	0 (0)	0 (0)	0 (0)	0 (0)	0 (0)	0 (0)	0 (0)
Diah and/or mel	0 (0)	0 (0)	0 (0)	0 (0)	0 (0)	0 (0)	0 (0)	0 (0)	0 (0)
Cough	0 (0)	0 (0)	0 (0)	0 (0)	0 (0)	0 (0)	0 (0)	0 (0)	0 (0)
Supp. NDs	0 (0)	0 (0)	0 (0)	0 (0)	0 (0)	0 (0)	0 (0)	0 (0)	0 (0)
Normal mms	20 (100)	20 (100)	20 (100)	40 (25)	120 (75)	160 (100)	0 (0)	10 (25)	40 (100)
Ab L/Ts	0 (0)	0 (0)	0 (0)	0 (0)	0 (0)	0 (0)	0 (0)	0 (0)	0 (0)
Ab Hs	0 (0)	0 (0)	0 (0)	0 (0)	0 (0)	0 (0)	0 (0)	0 (0)	0 (0)
Abnormal LNs	0 (0)	0 (0)	0 (0)	0 (0)	0 (0)	0 (0)	0 (0)	0 (0)	0 (0)
Rum Hypo	0 (0)	0 (0)	0 (0)	120 (75)	40 (25)	0 (0)	40 (100)	10 (25)	0 (0)
Cong Conj	0 (0)	0 (0)	0 (0)	120 (75)	40 (25)	0 (0)	40 (100)	10 (25)	0 (0)
Alopecia	0 (0)	0 (0)	0 (0)	0 (0)	0 (0)	0 (0)	0 (0)	0 (0)	0 (0)
Dehydration	0 (0)	0 (0)	0 (0)	120 (75)	40 (25)	0 (0)	40 (100)	10 (25)	0 (0)

^*^Number of buffalo-cows (%). Norm-Lab^gr^: Normal labored buffalo-cows. UtrTors^gr^: Mechanically corrected uterine torsed animals without medicament interference. UtrTors-Med^gr^: Mechanically corrected uterine torsed animals with medicament interference. Pre-calving^*^: 7 h pre-calving. Post-calving^*^: 48 h post-calving. Post-calving^**^: 96 h post-calving. CRT: Capillary refill time. Diah and/or mel: Diarrhoea and/or melena Supp. NDs: Suppurative Nasal discharge. MMs: mucous membranes. Ab L/Ts: Abnormal tracheal/lungs sound. Ab Hs: Abnormal heart sound LNs: lymph nodes. Rum Hypo: Ruminal hypomotility. Cong Conj: Congested conjunctiva. Reference values according to Jackson and Cockcroft [43]

Out of 160 buffalo-cow in UtrTors^gr^ (7 h pre-calving), 120 animals (75%) had fever (≥ 39.6 Ċ), polypnoea (≥ 30/m), tachycardia(≥ 90b/m), colicy pain, reduced rumen movements, injected eye capillaries and signs of dehydration. Moreover, normal appetite, mucous membranes and CRT, were reported in 40 (30%) of the diseased buffalo-cows in the same group pre-calving. 48 h post-calving, a clear improvement in these abnormal clinical findings were described whereas signs of fever, polypnoea, tachycardia, rumen hypomotility, congested conjunctiva and signs of dehydration were disappeared gradually post-calving (48 h post-calving; 25%) with manual correction of torsed uterus as they were reported in 25% of buffalo-cows in UtrTors^gr^ afterwards complete disappearance (100%) of these findings was reported at 96 h post-calving. Furthermore, normal appetite, mucous membranes and CRT were described in all buffaloes (n = 160; 100%) 96 h post-calving (Table 1).

At 7 h pre-calving in UtrTors-Med^gr^, signs of fever, polypnoea, tachycardia, colicy pain, reduced rumen movements, congested conjunctiva and dehydration were observed in all buffalo-cows (n = 40; 100%). In contrast, the normal appetite, mucous membranes and CRT (1–2 s), were not reported (n = 0; 0%) in these diseased buffalo-cows. 48 h post-calving, a clear improvement in these abnormal clinical findings were described whereas signs of fever, polypnoea, tachycardia, rumen hypomotility, congested conjunctiva and signs of dehydration were disappeared gradually post-calving (48 h post-calving) as a results to manual correction of torsed uterus as well as medicaments administration was conducted as these abnormal findings were reported in 25% of buffalo-cows in UtrTors-Med^gr^. Soon later, complete disappearance of these findings (0%) was reported at 96 h post-calving. Furthermore, normal appetite, mucous membranes and CRT were described in all buffaloes (n = 40; 100%) 96 h post-calving (Table 1).

Pain test (+ ve), emaciation, diarrhoea and/or melena, cough, suppurative nasal discharges, enlarged lymph nodes, abnormal heart and lung sounds and alopecia, were not observed in each of UtrTors^gr^ and UtrTors-Med^gr^, either 7 h pre-calving, 48 h post-calving or 96 h post-calving (Table 1).

No significant changes were stated between pre-calving or post-calving phases during the current study in Norm-Lab^gr^ either for temperature, pulse, respiration or rumen movements where they were within their reference values (Table 2).

**Table 2:** Mean values of temperature, pulse rate, respiratory rate and rumen movements in Norm-Lab^gr^ (n = 20), UtrTors^gr^ (n = 160) and UtrTors-Med^gr^ (n = 40) in buffalo-cows

	Norm-Lab^gr^			UtrTors^gr^			UtrTors-Med^gr^			Reference values
	Pre-calving^*^	Post-calving^*^	Post-calving^**^	Pre-calving^*^	Post-calving^*^	Post-calving^**^	Pre-calving^*^	Post-calving^*^	Post-calving^**^	
**Temperature (°C)**	37.83 ± 0.54^Ab^	38.11 ± 0.52^A1^	38.21 ± 0.74^ A¶^	39.74 ± 1.14^Aa^	38.52 ± 0.24^B1^	38.36 ± 0.61^B¶^	39.53 ± 0.62^Aa^	38.35 ± 0.24^B1^	38.44 ± 0.38^B¶^	(38.2 ± 0.64)^45^ or (37.8–39)^46^
**Pulse (Beat/min)**	64.22 ± 4.38^Ab^	66.42 ± 3.61^A1^	68.09 ± 2.43^ A¶^	86.06 ± 3.62^Aa^	70.33 ± 4.11^B1^	68.16 ± 4.66^B¶^	90.74 ± 4.82^Aa^	72.33 ± 2.66^B1^	70.06 ± 3.13^B¶^	(42–62)^44^ or (67 ± 10)^45^
**Respiration (/min)**	19.07 ± 2.11^Ab^	16.37 ± 1.75^A1^	18.26 ± 2.17^ A¶^	29.67 ± 3.11^Aa^	18.61 ± 2.03^B1^	20.08 ± 2.18^B¶^	33.73 ± 2.86^Aa^	20.07 ± 1.44^B1^	17.73 ± 2.08^B¶^	(22–28)^44^ or (15 ± 3)^45^
**Rumen (cycle/2mins)**	2.86 ± 1.04^Aa^	2.78 ± 0.81^A1^	2.92 ± 0.76^ A¶^	1.02 ± 0.62^Bb^	2.32 ± 0.44^A1^	2.63 ± 0.33^ A¶^	1.14 ± 0.61^Bb^	2.44 ± 0.34^A1^	2.78 ± 0.52^ A¶^	(3 ± 1)^45^

Norm-Lab^gr^: Normal labored buffalo-cows. UtrTors^gr^: Mechanically corrected uterine torsed animals without medicament interference. UtrTors-Med^gr^: Mechanically corrected uterine torsed animals with medicament interference. Pre-calving^*^: 7 h pre-calving. Post-calving^*^: 48 h post-calving. Post-calving^**^: 96 h post-calving. ^AB^Means within the same row with different superscript capital letters between different sampling hours either in Norm-Lab^gr^, UtrTors^gr^ or UtrTors-Med^gr^, were significantly different. ^ab^Means within the same row (Pre-calving^*^) with different superscript small letters between Norm-Lab^gr^, UtrTors^gr^ and UtrTors-Med^gr^, were significantly different. ^1^Means within the same row (Post-calving^*^) with different superscript numbers between Norm-Lab^gr^, UtrTors^gr^ and UtrTors-Med^gr^, were significantly different. ^¶^Means within the same row (Post-calving^**^) with different superscript symbols between Norm-Lab^gr^, UtrTors^gr^ and UtrTors-Med^gr^, were significantly different. Reference values according to Sajjad et al. [44]; Khalphallah et al. [45]; Rushdi et al. [46]

Values of temperature, pulse and respiration rates were significantly (p < 0.05) increased while rumen movements were significantly (p < 0.05) dropped during 7 h pre-calving phase in each of UtrTors^gr^ and UtrTors-Med^gr^ comparing with their values during 7 h pre-calving phase in Norm-Lab^gr^ whereas they were different from their reference intervals. These significant changes disappeared during post-calving phases (48 or 96 h post-calving) in each of UtrTors^gr^ and UtrTors-Med^gr^ when they compared with their values during the same post-calving phases in Norm-Lab^gr^ whereas they became within their reference ranges (Table 2).

Temperature, pulse and respiration rates were significantly (p < 0.05) reduced while rumen movements were significantly (p < 0.05) increased during post-calving phases (48 or 96 h post-calving) either in UtrTors^gr^ or in UtrTors-Med^gr^ comparing with their values during 7 h pre-calving phase either in UtrTors^gr^ or in UtrTors-Med^gr^ as they reached their reference intervals. No significant changes for these parameters were demonstrated between UtrTors^gr^ and UtrTors-Med^gr^ either during 48 h post-calving phase or during 96 h post-calving phase. These non-significant changes were reported between 48 h post-calving phase and 96 h post-calving phase either in UtrTors^gr^ or in UtrTors-Med^gr^ (Table 2).

### Complete blood picture

Except for TLC and DLC values, the whole blood picture parameters showed no remarkable changes between Norm-Lab^gr^, UtrTors^gr^ and UtrTors-Med^gr^ either during pre-caving or post-calving where they were within their reference values. No significant changes for these values were also reported between pre-caving and post-calving phases either in UtrTors^gr^ or in UtrTors-Med^gr^. In contrast, a significant (p < 0.05) increase in TLC and mature neutrophils while a significant (p < 0.05) drop in lymphocytes were stated during 7 h pre-calving phase in each of UtrTors^gr^ and UtrTors-Med^gr^ comparing with their values during the same pre-caving phase in Norm-Lab^gr^. These significant alterations in leucogram pictures were absent between Norm-Lab^gr^, UtrTors^gr^ and UtrTors-Med^gr^ either during 48 or 96 h post-calving phases. Leucogram pictures for TLC, neutrophils and lymphocytes had also other significant variations whereas a significant (p < 0.05) drop in TLC and neutrophils while a significant (p < 0.05) elevation in lymphocytes were observed in post-calving values comparing with pre-calving values either in UtrTors^gr^ or in UtrTors-Med^gr^ where they reached their reference values post-calving. These significant alterations in leukogram were absent between 48 and 96 h post-calving phases either in UtrTors^gr^ or in UtrTors-Med^gr^ (Table 3).

**Table 3:** Mean values of whole blood picture indices in Norm-Lab^gr^ (n = 20), UtrTors^gr^ (n = 160) and UtrTors-Med^gr^ (n = 40) in buffalo-cows

	Norm-Lab^gr^			UtrTors^gr^			UtrTors-Med^gr^			Reference values
	Pre-calving^*^	Post-calving^*^	Post-calving^**^	Pre-calving^*^	Post-calving^*^	Post-calving^**^	Pre-calving^*^	Post-calving^*^	Post-calving^**^	
**RBCs (**_**X**_ **10** ^**12**^ **/L)**	8.06 ± 1.24^Aa^	7.84 ± 1.62^A1^	8.24 ± 0.86^ A¶^	7.65 ± 0.89^Aa^	8.06 ± 1.33^A1^	8.12 ± 0.93^ A¶^	8.13 ± 1.12^Aa^	8.22 ± 0.88^A1^	8.18 ± 0.91^ A¶^	(5.2–8.4)^48^ or (7.54 ± 2.98)^49^
**PCV** **(L/L)**	0.34 ± 0.03^Aa^	0.32 ± 0.04^A1^	0.35 ± 0.02^ A¶^	0.36 ± 0.01^Aa^	0.38 ± 0.04^A1^	0.33 ± 0.03^ A¶^	0.33 ± 0.04^Aa^	0.35 ± 0.02^A1^	0.37 ± 0.02^ A¶^	(27.2–44.2)^48^ or (38 ± 3.24)^49^
**Hb** **(g/L)**	136.13 ± 6.48^Aa^	135.52 ± 4.63^A1^	133.08 ± 3.41^ A¶^	140.15 ± 6.48^Aa^	137.03 ± 5.11^A1^	136.35 ± 2.87^ A¶^	142.71 ± 3.07^Aa^	138.16 ± 3.88^A1^	139.26 ± 4.15^ A¶^	(95.3-145.04)^48^ or (118 ± 4.5)^49^
**TLCs (**_**X**_ **10** ^**9**^ **/L)**	7.82 ± 1.81^Ab^	8.18 ± 2.03^A1^	8.06 ± 1.73^ A¶^	16.11 ± 2.95^Aa^	8.06 ± 0.87^B1^	8.12 ± 1.04^B¶^	15.24 ± 1.22^Aa^	7.92 ± 1.03^B1^	8.33 ± 0.77^B¶^	(6–12.8)^48^ or (6.71 ± 1.63)^49^
**Neutrophiles (%)**	38.21 ± 2.08^Ab^	43.21 ± 2.74^A1^	41.53 ± 3.66^ A¶^	60.01 ± 4.11^Aa^	49.43 ± 2.74^B1^	45.13 ± 2.74^B¶^	58.34 ± 3.09^Aa^	46.07 ± 3.88^B1^	42.36 ± 4.15^B¶^	(31.67–53.91)^48^
**Lymphocytes (%)**	53.81 ± 4.11^Aa^	48.41 ± 3.88^A1^	48.34 ± 4.11^ A¶^	33.91 ± 3.43^Bb^	43.01 ± 3.88^A1^	46.24 ± 3.88^ A¶^	33.91 ± 5.18^Bb^	46.62 ± 4.35^A1^	47.16 ± 2.77^ A¶^	(41.66–56.8)^48^
**Monocytes (%)**	3.61 ± 1.04^Aa^	2.63 ± 0.71^A1^	5.16 ± 0.52^ A¶^	3.06 ± 0.76^Aa^	3.62 ± 0.71^A1^	3.83 ± 0.52^ A¶^	4.27 ± 1.02^Aa^	3.67 ± 1.02^A1^	4.81 ± 0.64^ A¶^	(0-7.81)^48^
**Eosinophiles (%)**	3.64 ± 1.33^Aa^	5.68 ± 1.42^A1^	4.28 ± 0.65^ A¶^	2.58 ± 1.14^Aa^	3.18 ± 1.42^A1^	4.08 ± 0.65^ A¶^	2.92 ± 0.36^Aa^	3.02 ± 0.65^A1^	5.04 ± 1.14^ A¶^	(0-8.59)^48^
**Band cells (%)**	0.73 ± 0.25^Aa^	0.83 ± 0.18^A1^	0.69 ± 0.29^ A¶^	0.52 ± 0.37^Aa^	0.76 ± 0.27^A1^	0.72 ± 0.29^ A¶^	0.56 ± 0.22^Aa^	0.62 ± 0.15^A1^	0.63 ± 0.33^ A¶^	(0-3.13)^48^ or (1.40 ± 0.52)^49^

Norm-Lab^gr^: Normal labored buffalo-cows. UtrTors^gr^: Mechanically corrected uterine torsed animals without medicament interference. UtrTors-Med^gr^: Mechanically corrected uterine torsed animals with medicament interference. Pre-calving^*^: 7 h pre-calving. Post-calving^*^: 48 h post-calving. Post-calving^**^: 96 h post-calving. RBCS: Red blood corpuscles. PCV: Packed cell volume. Hb: Haemoglobin concentration. TWBCs: Total leukocytic count. ^AB^Means within the same row with different superscript capital letters between different sampling hours either in Norm-Lab^gr^, UtrTors^gr^ or UtrTors-Med^gr^, were significantly different. ^ab^Means within the same row (Pre-calving^*^) with different superscript small letters between Norm-Lab^gr^, UtrTors^gr^ and UtrTors-Med^gr^, were significantly different. ^1^Means within the same row (Post-calving^*^) with different superscript numbers between Norm-Lab^gr^, UtrTors^gr^ and UtrTors-Med^gr^, were significantly different. ^¶^Means within the same row (Post-calving^**^) with different superscript symbols between Norm-Lab^gr^, UtrTors^gr^ and UtrTors-Med^gr^, were significantly different. Reference values according to Abd Ellah et al. [48]; Khalphallah et al. [49]

### Uterine torsion prevalence i.e. degree, rolling for treatment, duration and calves survival rates

The current study reported that out of 200 buffalo-cows with uterine torsion, 163 (81.5%) buffalo had uterine torsion of degree < 180^o^ while 37 (18.5%) animal had uterine torsion of degree > 180^o^. All buffalo-cows in UtrTors^gr^ (n = 160; 80%) had had uterine torsion of degree < 180^o^, hence, most of animals (n = 37; 18.5%) in UtrTors-Med^gr^ had uterine torsion of degree < 180^o^ and only 3 (1.5%) buffalo-cows had uterine torsion of degree < 180^o^ (Table 4).

**Table 4:** Degree of uterine torsion in UtrTors^gr^ (n = 160) and UtrTors-Med^gr^ (n = 40) in buffalo-cows

Degree of uterine torsion	No.		%	
**< 180** ^**o**^	163		81.5%	
	160 (UtrTors^gr^)	3 (UtrTors-Med^gr^)	80% (UtrTors^gr^)	1.5% (UtrTors-Med^gr^)
**> 180** ^**o**^	37 (UtrTors-Med^gr^)		18.5% (UtrTors-Med^gr^)	

UtrTors^gr^: Mechanically corrected uterine torsed animals without medicament interference. UtrTors-Med^gr^: Mechanically corrected uterine torsed animals with medicament interference

Mechanical rolling was a traditional technique for treatment of torsed uterus in buffaloes whereas this technique was successful in 160 (80%) buffalo-cows in UtrTors^gr^. Out of these 160 buffalo, mechanical correction of uterine torsion was successful with subsequent normal labor after 1 roll in 52 animals (32.5%), 2 rolls in 78 animals (48.75%), and 3 rolls in 30 animals (18.75%). This mechanical correction of torsed uterus was unsuccessful as well as subsequent calving was not induced whereas it required medicament interference in 40 (20%) buffalo-cows in UtrTors-Med^gr^ (Tables 5 and 6) as this medicinal interference after mechanical correction induced the vaginal delivery in this group.

**Table 5:** Successful roll for treatment in buffalo-cows with uterine torsion (n = 200)

successful rolling	No.	%
**Successful**	160 (UtrTors^gr^)	80% (UtrTors^gr^)
**Unsuccessful**	40 (UtrTors-Med^gr^)	20% (UtrTors-Med^gr^)

UtrTors^gr^: Mechanically corrected uterine torsed animals without medicament interference. UtrTors-Med^gr^: Mechanically corrected uterine torsed animals with medicament interference

**Table 6:** Numbers of successful rolls for treatment in UtrTors^gr^ (n = 160) buffalo-cows

Numbers of rolling for treatment	No.	%
**1 Roll**	52	32.5%
**2 Rolls**	78	48.75%
**3 Rolls**	30	18.75%

UtrTors^gr^: Mechanically corrected uterine torsed animals without medicament interference

Regarding to the duration of uterine torsion until either mechanical final correction was conducted or medicaments interference plus the mechanical correction was used, was variable whereas the duration of uterine torsion was 0–24 h, 24–48 h, 48–72 h and > 72 h in 16 (8%), 42 (21%), 60 (30%) and 82 (41%) buffalo-cows with torsed uterus, respectively. This revealed that the required time for correction of uterine torsion was the shortest (0–24 h) in few numbers 16 (8%) of buffaloes, however, most of animals (71%) needed longer time (48 to > 72 h) until correction of torsed uterus was conducted in were corrected (Table 7).

**Table 7:** Duration of torsion (n = 200) in buffalo-cows with uterine torsion (n = 200)

Duration of torsion	No.	%
**0–24 h**	16	8%
**24–28 h**	42	21%
**28–72 h**	60	30%
**> 72**	82	41%

The survival rates of newly born buffalo-calves (n = 200) in buffaloes with uterine torsion in the two investigated groups included 155 (77.5%) dead calf and 45 (22.5%) alive one (Table 8).

**Table 8:** The survival rate of newly born buffalo-calves (n = 200) in buffalo-cows with uterine torsion

The survival rate of the calves	No.	%
**Dead**	155	77.5%
**Alive**	45	22.5%

### Buffalo-calf birth weight and placental characterization

Buffalo-calves birth weights were significantly (p < 0.05) higher in Norm-Lab^gr^ than those in UtrTors^gr^ and UtrTors-Med^gr^. These significant changes were not reported between UtrTors^gr^ and UtrTors-Med^gr^ (Table 9).

**Table 9:** Mean values of buffalo-calf birth weights and placental characterization in Norm-Lab^gr^ (n = 20), UtrTors^gr^ (n = 160) and UtrTors-Med^gr^ (n = 40) in buffalo-cows

	Norm-Lab^gr^	UtrTors^gr^	UtrTors-Med^gr^
**Calf birth weight (kg)**	39.944 ± 0.423^a^	38.842 ± 0.107^b^	38.908 ± 0.122^b^
**Pl weight (kg)**	5.564 ± 0.121^b^	5.857 ± 0.199^a^	5.825 ± 0.151^a^
**TCW (kg)**	1.999 ± 0.018^a^	1.897 ± 0.015^b^	1.889 ± 0.0179^b^
**TCN**	89.1 ± 1.197^a^	87.1 ± 1.287^b^	87.2 ± 0.789^b^
**ALCL (cm)**	8.39 ± 0.458^a^	8.09 ± 0.661^a^	8.1 ± 0.489^a^
**AMCL (cm)**	6.58 ± 0.476^a^	6.28 ± 0.397^a^	6.240 ± 0.659^a^
**ASCL (cm)**	4.06 ± 0.443^a^	3.76 ± 0.572^b^	3.81 ± 0.648^b^
**ALCW (cm)**	9.07 ± 0.319^a^	8.49 ± 0.681^b^	8.52 ± 0.592^b^
**AMCW (cm)**	6.825 ± 0.251^a^	6.265 ± 0.504^b^	6.242 ± 0.563^b^
**ASCW (cm)**	4.46 ± 0.35^a^	3.91 ± 0.321^b^	3.83 ± 0.33^b^
**ALCD (cm)**	0.98 ± 0.042^a^	0.94 ± 0.084^a^	0.96 ± 0.07^a^
**AMCD (cm)**	0.980 ± 0.092^a^	0.930 ± 0.125^a^	0.920 ± 0.123^a^
**ASCD (cm)**	0.715 ± 0.072^a^	0.564 ± 0.125^b^	0.572 ± 0.126^b^
**PE**	7.183 ± 0.192^a^	6.638 ± 0.217^b^	6.683 ± 0.152^b^
**CD**	0.016 ± 0.0005^a^	0.0149 ± 0.0006^b^	0.015 ± 0.0005^b^

Norm-Lab^gr^: Normal labored buffalo-cows. UtrTors^gr^: Mechanically corrected uterine torsed animals without medicament interference. UtrTors-Med^gr^: Mechanically corrected uterine torsed animals with medicament interference. Pl weight: Placental weight. TCW: Total cotyledon weight. TCN: Total cotyledon number. ALCL: Average large cotyledon length. AMCL: Average medium cotyledon length. ASCL: Average small cotyledon length. ALCW: Average large cotyledon width. AMCW: Average medium cotyledon width. ASCW: Average small cotyledon width. ALCD: Average large cotyledon depth. AMCD: Average medium cotyledon depth. ASCD: Average small cotyledon depth. PE: Placental efficiency. CD: Cotyledon density. PE = calf birth weight/placental weight per kg. CD = number of cotyledon/ placental weight per gram. ^ab^Means within the same row with different superscript capital letters between Norm-Lab^gr^, UtrTors^gr^ and UtrTors-Med^gr^, were significantly different

Some of placental characterization parameters showed significant alterations through the present work. Most of these significant variations were higher in Norm-Lab^gr^ comparing with UtrTors^gr^ and UtrTors-Med^gr^ except for PI weight. PI weights were significantly increased in UtrTors^gr^ and UtrTors-Med^gr^, comparing with those in Norm-Lab^gr^. Significant elevations in values of TCW, TCN, ASCL, ALCW, AMCW, ASCW, ASCD, PE and CD, were demonstrated in Norm-Lab^gr^ comparing with their values in UtrTors^gr^ and UtrTors-Med^gr^. These significant changes were absent between UtrTors^gr^ and UtrTors-Med^gr^. In contrast, other placental characterization parameters including ALCL, AMCL, ALCD and AMCD had no remarkable changes between Norm-Lab^gr^, UtrTors^gr^ and UtrTors-Med^gr^ (Table 9).

### Buffalo-cow’s milk composition and somatic cell count (SCC)

Buffalo-cow’s milk composition including milk proteins, fat, solids not fat (SNF), lactose and total solids, had no significant variations between Norm-Lab^gr^, UtrTors^gr^ and UtrTors-Med^gr^ where they were within their reference intervals (Table 10).

**Table 10:** Mean values of buffalo-cow’s milk composition and somatic cell count (SCC) in Norm-Lab^gr^ (n = 20), UtrTors^gr^ (n = 160) and UtrTors-Med^gr^ (n = 40)

	Norm-Lab^gr^	UtrTors^gr^	UtrTors-Med^gr^	Reference values
Protein %	4.6 ± 0.15^a^	4.41 ± 0.21^a^	4.33 ± 0.17^a^	(3.60–3.85)^57^ or (3.77 ± 0.26)^58^
Fat %	6.87 ± 0.23^a^	6.35 ± 0.11^a^	6.45 ± 0.15^a^	(7.0–7.2)^57^ or (7.3 ± 0.5)^58^
Milk SNF %	10.95 ± 0.54^a^	10.76 ± 0.46^a^	10.55 ± 0.68^a^	(9.8–10.1)^57^ or (9.2 ± 0.2)^58^
Lactose %	5.82 ± 0.04^a^	5.52 ± 0.07^a^	5.71 ± 0.09^a^	(4.99–5.24)^57^ or (4.76 ± 0.18)^58^
Total solids %	17.65 ± 0.03^a^	17.28 ± 0.08^a^	18.04 ± 0.06^a^	(16.9–17.4)^57^ or (16.7 ± 0.1)^58^
pH	6.3 ± 0.06^a^	6.4 ± 0.07^a^	6.5 ± 0.05^a^	(6.7 to 6.9)^59^
Milk SCC (_X_ 10^5^ cells/ml)	1.69 ± 0.13^a^	1.72 ± 0.18^a^	1.78 ± 0.14^a^	(3.21 ± 0.179)^60^ or (0.39–1.76)^61^

Norm-Lab^gr^: Normal labored buffalo-cows. UtrTors^gr^: Mechanically corrected uterine torsed animals without medicament interference. UtrTors-Med^gr^: Mechanically corrected uterine torsed animals with medicament interference. Milk SNF: milk solids not fat. Milk SCC: Somatic cell count. ^a^Means within the same row with different superscript capital letters between Norm-Lab^gr^, UtrTors^gr^ and UtrTors-Med^gr^, were significantly different. Reference values according to Abd El-Salam and El-Shibiny [57]; Khan et al. [58]; Singh and Ludri [59]; Ahmad et al. [60]; Patil et al. [61]

Milk pH values were not significantly altered between Norm-Lab^gr^ and uterine torsed buffalo-cows. They were within their reference ranges (Table 10).

Milk SCC values had no significant changes between Norm-Lab^gr^, UtrTors^gr^ and UtrTors-Med^gr^ as they were within their reference values (Table 10).

## Discussion

### Clinical findings

The severity of the symptoms was greatly influenced by the degree of torsion, and clinical symptoms might not appear with torsion below 180^0^. Due to the narrowing of the birth canal, which prevented the foetus from entering the pelvis, which was required for normal abdominal straining, abdominal straining was typically linked with second stage labor, and any indications associated with this stage of labor were missing or minor [42]. The present work reported that buffalo-cows with uterine torsion had variable signs 7 h pre-calving either in UtrTors^gr^ or in UtrTors-Med^gr^ including anorexia, prolonged CRT i.e. >1–2 s; as mentioned by Jackson and Cockcroft [43], fever, polypnoea, tachycardia, colicy pain, reduced rumen movements, congested mucous membranes and conjunctiva and dehydration. These findings were more prominent in all buffaloes in UtrTors-Med^gr^ while they were observed in most buffaloes in UtrTors^gr^. A clear improvement in these abnormal clinical manifestations had been observed 2 days post-calving whereas their complete disappearance had been demonstrated in both two groups of uterine torsion buffaloes 4 days post-calving. Moreover, Robert [21] mentioned that in severe cases of torsed uterus in which blood supply to and from the uterus was severely restricted, signs might include foetid diarrhea, subnormal body temperature, expiratory grunt, weak pulse, cold extremities, shock and collapse within 24–72 h in some animals [21]. In contrast, the present study revealed absence of pain test (+ ve), emaciation, diarrhoea and/or melena, cough, suppurative nasal discharges, enlarged lymph nodes, abnormal heart and lung sounds and alopecia in each of UtrTors^gr^ and UtrTors-Med^gr^, either 7 h pre-calving, 48 h post-calving or 96 h post-calving. Furthermore, significant changes in rectal temperature, heart and respiratory rates and rumen movement in buffalo with uterine torsion through the present work whereas significant elevations in temperature and pulse and respiratory rates while a significant drop in rumen motility were more clearful in UtrTors^gr^ and UtrTors-Med^gr^ comparing with their values during 7 h pre-calving phase in Norm-Lab^gr^ whereas they were they were different from their reference intervals mentioned by Sajjad et al. [44]; Khalphallah et al. [45]; Rushdi et al. [46]. These significant changes disappeared between UtrTors^gr^, UtrTors-Med^gr^ and Norm-Lab^gr^, during post-calving phases (48 or 96 h post-calving) whereas they became within their reference ranges. On other hand, as a result to correction and/or medicaments interference a clear reduction in body temperature, pulse and respiration rates as well as remarkable elevation in rumen movements were stated during post-calving phases (48 or 96 h post-calving) either in UtrTors^gr^ or in UtrTors-Med^gr^ comparing with those during 7 h pre-calving phase either in UtrTors^gr^ or in UtrTors-Med^gr^ whereas they reached their reference intervals. Ali et al. [5] confirmed the current result as they indicated the main clinical indications of uterine torsion in buffalo-cows included straining or colic for prolonged time, reduction in feed intake and constipation in 88/126 (69.8%), 72/126 (57.1%) and 13/126 (10.3%) of the cases, respectively. From the time the clinical signs first appeared until it was treated, the average time for torsion was 20 to 168 h.

### Complete blood picture

In buffalo-cows with uterine torsion, a normocytic normochromic anaemia was frequently found by total blood count. In some instances, though, leukocytosis with neutrophilia and monocytosis might also be seen [47]. Regarding to the current work with except for TLC and DLC values, the whole blood picture parameters showed no remarkable changes between Norm-Lab^gr^, UtrTors^gr^ and UtrTors-Med^gr^ either during pre-caving or post-calving where they were within their reference values mentioned by Abd Ellah et al. [48]; Khalphallah et al. [49]. No significant changes for these values were also reported between pre-caving and post-calving phases either in UtrTors^gr^ or in UtrTors-Med^gr^. In contrast, a significant increase in TLC and neutrophils while a significant drop in lymphocytes were stated during 7 h pre-calving phase in each of UtrTors^gr^ and UtrTors-Med^gr^ comparing with their values during the same pre-caving phase in Norm-Lab^gr^. These significant alterations in leucogram pictures were absent between Norm-Lab^gr^, UtrTors^gr^ and UtrTors-Med^gr^ either during 48 or 96 h post-calving phases. Leucogram pictures for TLC, neutrophils and lymphocytes had also other significant variations whereas a significant drop in TLC and neutrophils while a significant elevation in lymphocytes were observed in post-calving values comparing with pre-calving values either in UtrTors^gr^ or in UtrTors-Med^gr^. These significant alterations in leukogram were absent between 48 and 96 h post-calving phases either in UtrTors^gr^ or in UtrTors-Med^gr^. Moreover, the earlier articles also said that a complete blood count might be useful in figuring out the severity of a problem and its prognosis. While band cells appeared as a result of certain infected buffaloes’ toxemic state, an increase in monocytes frequently found in these cases indicated a long-standing uterine infection [50, 51]. Blood losses from severely twisted uterine blood arteries may be the cause of the observed hypochromic anaemia, which is characterised by a decreased mean corpuscular haemoglobin content [5].

### Uterine torsion prevalence i.e. degree, rolling for treatment, duration and calves survival rates

The current study reported that out of 200 buffalo-cows with uterine torsion, 81.5% of buffalo-cows had uterine torsion of degree < 180^o^ as most of them belonged to UtrTors^gr^ (n = 160; 80%) while 18.5% of animals had uterine torsion of degree > 180^o^ as most of them belonged to UtrTors-Med^gr^ (n = 37; 18.5%). These findings were relatively supported by Aubry et al. [24]. In contrast, Zaher et al. [25] stated that most uterine torsion cases (25–35) were severe (270–360^o^), whereas a few cases (six) were moderate (180–270o), and just a few cases (four) were mild (180^o^). They also observed that uterine torsion ranged from 180 to 270 degrees in 75% of cases, and that torsion greater than 360 degrees was extremely uncommon (9%). According to Satish et al. [22], just one case (1.81%) was affected with a maximum degree of 360° of uterine torsion, whereas 49.09% (27/55) of animals demonstrated uterine torsion of 90–180° and the same percentage of animals demonstrated uterine torsion of 180–360°. According to Zaher et al. [25], the degree of uterine torsion varied from animal to animal, and this could be explained by a variety of factors including increased uterine mobility, excessive foetal movement, large foetus size, anatomical placement of the uterus between the rumen, intestine, and abdominal wall, sudden animal slip, amount of tension, and length of broad ligament. Additionally, the volume of the foetal fluid. Moreover, buffaloes’ weak abdominal muscles and greater abdominal size allowed the uterus to rotate freely [25]. Moreover, the cephalic region of the vagina, which caused stenosis and spiral twisting of its wall, was involved in the majority of cases of uterine torsion [21].

If the dam was recumbent and the foetus could not be approached because of the severity of the torsion or if the torsion developed before the anticipated time of delivery, rolling was suggested [21]. Mechanical rolling through the current study was a traditional technique for treatment of torsed uterus in buffaloes whereas this technique was successful in 80% of buffalo-cows in UtrTors^gr^. Out of these 160 buffalo, mechanical correction of uterine torsion was successful with subsequent normal labor after 1 roll in 32.5% of animals, 2 rolls in 48.75% of animals, and 3 rolls in 18.75% of buffalo-cows. Ali et al. [5] reported that 51.6% of buffalo-cow uterine torsion cases resulted in vaginal delivery when the mother was slowly rolled. According to Zaher et al. [25], 94.28% of the treated animals had a rolling success rate. On the other hand, Frazer et al. [39] reported that upon trans-rectal palpation, the orientation of the broad ligaments was noticeably altered depending on whether the torsion was to the left or right, resulting in the appropriate wide ligament being pulled tightly across the uterus. Mostly torsion cases involved left side in counter clockwise direction with 45 to 90^0^ torsion being very uncommon, 20% were 90-180^0^, 57% were 180–270^0^ and 22% were 270–360^0^. Successful trials to roll the buffalo-cows and deliver fetus reduced when the severity of uterine torsion increased [39]. Circulatory disorders might cause both the foetus and cow to die if quick diagnosis and treatment were not performed, and expulsion of the foetus was impossible unless the disease was corrected [52]. The majority of uterine torsions never required surgery, and caesarean sections were seldom the first option. In order to prevent unnecessary stress from rolling, delayed uterine torsion (> 72 h) should be immediately submitted to a caesarean procedure [6]. An animal with an emphysematous foetus in a friable, infected uterus was not a good candidate for abdominal surgery [47]. When deciding on a caesarean, the expense of the procedure and the worth of the animal should be carefully evaluated [26].

If torsion still existed after three rolls, it was recommended to acknowledge defeat and consider surgery [53, 54]. This might support the findings of the present work when the mechanical correction of torsed uterus was unsuccessful as well as subsequent calving or vaginal delivery was not induced whereas it required medicament interference in all buffalo-cows in UtrTors-Med^gr^ (n = 40; 20%) to induce vaginal delivery. Furthermore, Ali et al. [5] revealed that in some of these cases (9.5%), a caesarean section was carried out on buffalo-cows with uterine torsion after unsuccessful detorsion attempts or as a result of failure of the cervix dilation after successful mechanical correction of the torsed uterus. Here, a large percentage of caesarean sections were performed right away (38.9%). In some of these cases (9.5%), a caesarean section was carried out on buffalo-cows with uterine torsion after unsuccessful detorsion attempts or as a result of failure of the cervix dilation after successful mechanical correction of the torsed uterus. Here, a large percentage of caesarean sections were performed right away (38.9%).

According to Schönfelder and Hasenclever [7], future fertility was inversely linked with both the severity and extent of torsion. Based on the severity of the vascular compromise, the length of the torsion, and the quick, accurate diagnosis that was followed by the skillful, precise manipulation of a clinician, successful outcomes of torsion-affected cattle were determined [13]. Regarding to the duration of uterine torsion in the present work until either mechanical final correction was conducted or medicaments interference plus the mechanical correction was used, was variable whereas the duration of uterine torsion was 0–24 h, 24–48 h, 48–72 h and > 72 h in 8%, 21%, 30% and 41% of buffalo-cows with torsed uterus, respectively. This revealed that the required time for correction of uterine torsion was the shortest (0–24 h) in few numbers of buffaloes (n = 16; 8%), however, most of animals (71%) needed longer time (48 to > 72 h) until correction of torsed uterus was conducted in were corrected. Furthermore, according to various reports, depending on whether this investigation was based on a field research or hospital referral states, the mortality rates of dams in cows ranged from 5 to 30% [39, 47, 55]. The progression of uterine oedema and obvious ischemia necrosis was influenced by the severity of the uterus torsion and the length of the vascular compromise [38].

The survival rates of newly born buffalo-calves in buffalo-cows with uterine torsion in the two investigated groups included 77.5% dead calves (n = 155) and 22.5% (n = 45) alive one. On the other hand, in cases of uterine torsion in buffaloes, the severity and duration of the torsion as well as the stage of gestation were risk factors affecting foetal mortality [5]. A factor threatening foetal life in severe torsion was undoubtedly the degree of uterine vascular compression [55]. Due to the possibility that hypoxia could occur from placental separation, delayed detection of these disorders almost always led to the delivery of a dead foetus [39, 55]. When compared to cows, buffaloes had a higher frequency of foetal fatalities due to uterine torsion [39, 47, 55]. This could be explained by the fact that in buffaloes, severe uterine torsion predominated and developed before term [5]. The middle uterine artery was compressed as the degree of torsion increased, which reduced the amount of oxygen reaching the foetus [56]. The twisted or torsed uterus’s restricted venous outflow and arterial perfusion led to ischemia, hypoxia, and cell death, which resulted in irreparable significant damage to the myometrium and endometrium and ultimately the loss of the foetus [26].

### Buffalo-calf birth weight and placental characterization

Buffalo-calves birth weights were significantly higher in Norm-Lab^gr^ than those in UtrTors^gr^ and UtrTors-Med^gr^. These significant changes were not reported between UtrTors^gr^ and UtrTors-Med^gr^.

Some of placental characterization parameters showed significant alterations through the present work. Most of these significant variations were higher in Norm-Lab^gr^ comparing with UtrTors^gr^ and UtrTors-Med^gr^ except for PI weight. PI weights were significantly increased in UtrTors^gr^ and UtrTors-Med^gr^, comparing with those in Norm-Lab^gr^. Significant elevations in values of TCW, TCN, ASCL, ALCW, AMCW, ASCW, ASCD, PE and CD, were demonstrated in Norm-Lab^gr^ comparing with their values in UtrTors^gr^ and UtrTors-Med^gr^. These significant changes were absent between UtrTors^gr^ and UtrTors-Med^gr^. In contrast, other placental characterization parameters including ALCL, AMCL, ALCD and AMCD had no remarkable changes between Norm-Lab^gr^, UtrTors^gr^ and UtrTors-Med^gr^.

#### Buffalo-cow’s milk composition and somatic cell count (SCC)

Berry et al. [29]; Gaafar et al. [30] noted decreased average daily milk yield and overall milk yield in dystocia-affected cows compared to normal cows, however they did not investigate its impact on milk composition. Referring to the present study, buffalo-cow’s milk composition including milk proteins, fat, SNF, lactose and total solids, had no significant variations between Norm-Lab^gr^, UtrTors^gr^ and UtrTors-Med^gr^ where they were within their reference intervals mentioned by Abd El-Salam and El-Shibiny [57]; Khan et al. [58]. Furthermore, values of milk pH values and Milk SCC were also not significantly changed between Norm-Lab^gr^ and uterine torsed buffalo-cows. They were within their reference ranges mentioned by Singh and Ludri [59]; Ahmad et al. [60]; Patil et al. [61].

## Conclusion

The study concluded pre-calving remarkable variations in clinical findings, leukogram picture, calf birth weight and some placental characterization parameters between Norm-Lab^gr^ and each of UtrTors^gr^ and UtrTors-Med^gr^ whereas these variations disappeared post-partum as a result to either only mechanical correction or mechanical correction plus medicaments interference. No pre-or post-calving significant changes between UtrTors^gr^ and UtrTors-Med^gr^ except for the abnormal clinical findings were more representative in UtrTors-Med^gr^ than those in UtrTors^gr^ particularly pre-calving. The applied pre-calving therapeutic regimen including dexamethasone-prostaglandin-receptal combination had a powerful potential efficacy that induced vaginal delivery of calves in UtrTors-Med^gr^ as well as prepartum mechanical correction of torsed uterus approved higher efficacy in UtrTors^gr^. The applied prepartum mechanical correction of torsed uterus and/or pre-calving therapeutic regimen as well as subsequent post-calving, post uterine correction applied medicament treatment accelerated rapid recovery of affected buffalo-cows through achieving rapid restoring of their physiological parameters. Buffalo-cow’s milk composition, milk pH and milk SCC were not affected whereas no significant variations were reported between Norm-Lab^gr^, UtrTors^gr^ and UtrTors-Med^gr^.

.

## Data Availability

The datasets used and/or analyzed during the current study are available from the corresponding author on reasonable request. Data was also available after publishing in this journal.

## Abbreviations

ALCL: average large cotyledon length
ALCD: Average large cotyledon depth
ALCW: average large cotyledon width
AMCD: average medium cotyledon depth
AMCL: average medium cotyledon length
AMCW: average medium cotyledon width
ASCD: average small cotyledon depth
ASCL: average small cotyledon length
ASCW: average small cotyledon width
CD: cotyledon density
CRT: capillary refill time
DLC: differential leukocytic count
Hb: haemoglobin
Norm-Labgr: normal labored buffalo-cows
PCV: packed cell volume
PE: Placental efficiency
Pl weight: placental weight
RBCs: red blood corpuscles
SCC: somatic cell count
SNF: solids not fat
TCN: total cotyledon number
TLC: total leucocytic count
TMR: total mixed ration
TCW: total cotyledon weight
UtrTorsgr: mechanically corrected uterine torsed animals without medicament interference
UtrTors-Medgr: mechanically corrected uterine torsed animals with medicament interference
